# Monitoring Mitochondrial Partial Oxygen Pressure During Cardiac Arrest and Extracorporeal Cardiopulmonary Resuscitation. An Experimental Pilot Study in a Pig Model

**DOI:** 10.3389/fcvm.2021.754852

**Published:** 2021-10-25

**Authors:** Loes Mandigers, Jan-Steffen Pooth, Mark A. Wefers Bettink, Corstiaan A. den Uil, Domagoj Damjanovic, Egbert G. Mik, Sam Brixius, Diederik Gommers, Georg Trummer, Dinis dos Reis Miranda

**Affiliations:** ^1^Department of Intensive Care, Erasmus University Medical Center, Rotterdam, Netherlands; ^2^Department of Cardiovascular Surgery, Heart Center Freiburg University, Faculty of Medicine, University of Freiburg, Freiburg, Germany; ^3^Department of Anaesthesiology, Erasmus University Medical Center, Rotterdam, Netherlands; ^4^Department of Cardiology, Erasmus University Medical Center, Rotterdam, Netherlands; ^5^Department of Intensive Care, Maasstad Hospital, Rotterdam, Netherlands

**Keywords:** heart arrest, cardiac arrest, extracorporeal cardiopulmonary resuscitation, mitochondrial oxygen pressure, circulation monitoring

## Abstract

**Introduction:** Ischemia and reperfusion are crucial in determining the outcome after cardiac arrest and can be influenced by extracorporeal cardiopulmonary resuscitation (ECPR). The effect of ECPR on the availability and level of oxygen in mitochondria remains unknown. The aim of this study was to find out if skin mitochondrial partial oxygen pressure (mitoPO_2_) measurements in cardiac arrest and ECPR are feasible and to investigate its course.

**Materials and Methods:** We performed a feasibility test to determine if skin mitoPO_2_ measurements in a pig are possible. Next, we aimed to measure skin mitoPO_2_ in 10 experimental pigs. Measurements were performed using a cellular oxygen metabolism measurement monitor (COMET), at baseline, during cardiac arrest, and during ECPR using the controlled integrated resuscitation device (CIRD).

**Results:** The feasibility test showed continuous mitoPO_2_ values. Nine experimental pigs could be measured. Measurements in six experimental pigs succeeded. Our results showed a delay until the initial spike of mitoPO_2_ after ECPR initiation in all six experimental tests. In two experiments (33%) mitoPO_2_ remained present after the initial spike. A correlation of mitoPO_2_ with mean arterial pressure (MAP) and arterial partial oxygen pressure measured by CIRD (CIRD-PaO_2_) seemed not present. One of the experimental pigs survived.

**Conclusions:** This experimental pilot study shows that continuous measurements of skin mitoPO_2_ in pigs treated with ECPR are feasible. The delay in initial mitoPO_2_ and discrepancy of mitoPO_2_ and MAP in our small sample study could point to the possible value of additional measurements besides MAP to monitor the quality of tissue perfusion during cardiac arrest and ECPR.

## Introduction

In cardiac arrest, the duration of ischemia is an important determinant for survival and neurological outcome (1, 2). In order to shorten this ischemic period during cardiac arrest, extracorporeal cardiopulmonary resuscitation (ECPR) can be used to recover circulation and effective oxygen transport. The possible beneficial effect of ECPR on neurologically favorable survival has already been studied previously (3). However, the best treatment protocol of ECPR regarding ECPR settings is still unknown.

To determine if the recovery of circulation and oxygen delivery using ECPR are sufficient, we measured oxygen in the mitochondria, as final destination of oxygen. After all, mitochondria are important for generating energy, using oxygen, for cellular processes and maintaining life (4, 5). Protoporphyrine IX (PpIX) is an endogenously present porphyrin in the mitochondria, which can be enhanced by administrating 5-aminolevulinic acid hydrochloride (ALA) crème (6). The subcellular distribution of PpIX in ALA stimulated cells has been studied using wide-field fluorescence microscopy (7). Previous research has shown the possibilities of measuring partial oxygen pressure (PO_2_) in the mitochondria by PpIX using its oxygen-dependent delayed fluorescence (7–10). To confirm that this delayed fluorescence truly measures inside the mitochondria, a previous study compared photobleaching (a contrast enhancement technique for PpIX) to MitoTracker Green (a method to identify mitochondria). This comparison showed a high degree of co-localization (7). This confirmed that, for a time window of several hours after ALA administration, PpIX measurements with delayed fluorescence corresponds to a mitochondrial localization (7). This method of measuring mitochondrial PO_2_ (mitoPO_2_) has also been validated to perform well in the skin (11). In addition, the possibility to perform continuous mitoPO_2_ measurements is shown in adults by Ubbink et al. (10) and in newborns by Costerus et al. (12).

The primary aim of this study is to find out if continuous measurements of skin mitoPO_2_ in a pig are feasible and what the course of this mitoPO_2_ is during cardiac arrest and ECPR. The secondary aims are to investigate if there is a correlation between the course of mitoPO_2_ and mean arterial pressure (MAP) and to identify the correlation between the course of mitoPO_2_ and favorable neurological survival.

## Methods

Between November 2017 and September 2020, 11 male and female German landraces pigs (weight: 50.0–82.0 kg) were eligible for skin mitoPO_2_ measurements. Continuous skin mitoPO_2_ measurements in pigs with experimental settings of cardiac arrest treated with ECPR have not been performed before. In order to determine if these measurements are possible to perform and feasible in our test set up we first performed a feasibility test in one pig. This test showed that continuous measurements in this set up was possible. Therefore, we selected 10 pigs to perform continuous skin mitoPO_2_ measurements as experimental test group. Of these 10 experimental tests, one could not be performed due to technical failure before the start of the test.

All animals received humane care and were treated in compliance with the Guide for the Care and Use of Laboratory Animals published by the US National Institutes of Health (13). The experiments were performed in accordance with the rules and regulations of the German animal protection law and the animal care guidelines of the European Community. The experiments were performed in the University of Freiburg, a highly experienced animal lab performing many ECPR procedures in pigs, and approved by the committee for ethics of the University Hospital Freiburg, Freiburg, Germany (no.G-15/148).

### Preparation of Tests

After premedication (20 mg/kg ketamine and 0.5 mg/kg midazolam) an intravenous (IV) access was placed, the pigs were sedated and paralyzed (3–4 mg/kg propofol and 0.2 mg/kg vecuronium), intubated, and mechanically ventilated. Continuous intravenous anesthesia consisted of the administration of 10–15 mg/kg/h propofol, 1–5 μg/kg/h fentanyl and 0.2–0.4 mg/kg/h vecuronium. In the pre-arrest period the core temperature we aimed for was 36–38°C. This temperature was measured by a nasal temperature sensor and in case of a low body temperature the pig was heated using a warming blanket.

In order to perform mitoPO_2_ measurements, a part of the skin (~1 cm^2^) was prepared. First, hair was removed by shaving, the skin was roughened, and then the skin was cleaned using sodium chloride (NaCl 0.9%) and ethanol (70%). The 20% ALA crème was prepared by mixing 400 mg ALA (Fagron, Barsbüttel, Gemany) with 2 g Lanettecrème I FNA (Teva Nederland BV, Haarlem NL) (6). To avoid photobleaching of PpIX by light, the applied ALA crème was directly covered by a plaster and by aluminum foil. The crème was placed 3 h before the first measurement and during this waiting time it was continuously protected to light (7, 11). Because of the use of a mechanical cardiopulmonary resuscitation (CPR) device and cannulation in the right groin and neck, we measured the mitoPO_2_ in the left axilla/neck region. Five minutes before induction of arrest, the effect of the ALA crème was tested by compression of the sensor on the skin (8, 10). When the measurements were finished, the skin was again covered to protect from light in order to protect it from burn lesions.

### Test Procedure

The protocols and set up of the feasibility test and the experimental tests were slightly different, we will describe the test procedures separately.

#### Feasibility Test

Ventricular fibrillation (VF) was induced by electrical stimulation *via* a Swan-Ganz catheter (Edwards Lifesciences Corp., Irvine, CA, USA). During 20 min of cardiac arrest, venous and arterial access was surgically generated via the right external jugular vein (23 Fr cannula) and the right common femoral artery (17 Fr cannula), respectively. In the period of cardiac arrest, mechanical ventilation was stopped and no life support was applied. After 20 min of cardiac arrest, ECPR was initiated with blood flow varying from 5.9 to 7.6 L/min. External defibrillation was performed in case of persisting VF. ECPR was weaned and stopped 60 min after initiation. If the animal could be weaned from ECPR, it was subsequently weaned from the ventilator and transferred to the animal facility after extubation. The pig was examined daily and neurological outcome was tested using a modified species-specific neurological deficit score (NDS) (14). This NDS ranges from 0 (normal) to 500 (brain death) and a favorable outcome was defined as NDS below 50 (14, 15). Euthanasia was performed in tabula in case the pig could not be weaned off extracorporeal circulation or invasive ventilation, in case the pig was expected to have inhumane suffering or prolonged death (an NDS of >200 at 24 h or an NDS of >120 at 48 h), and otherwise after 7 days (15).

#### Experimental Tests

In the nine experimental tests, the ECPR implantation and induction of VF was the same as described at the feasibility test, except the timing of ECPR cannulation. In the experimental tests, this was already performed in the pre-arrest period. Next, after 5 min of VF, basic life support (BLS) was started with cardiopulmonary resuscitation (CPR) using a mechanical compression device (Corpuls CPR, GS Elektromedizinische Geräte G. Stemple GmbH, Kaufering, Germany) for 8 min. The next 22 min consisted of advanced life support (ALS), with additional administration of epinephrine every 4 min. After 35 min of cardiac arrest and CPR, ECPR was initiated with blood flow varying from 5.5 to 7.9 L/min and a 20 ml bolus of 7.45% potassium was applied for rhythm conversion. During ECPR in case of persisting VF, the heart was electrically defibrillated. If the VF sustained after three defibrillations, amiodarone and lidocaine were administered. If needed, continuous norepinephrine was administered with an aimed MAP of 60–80 mmHg. For these 10 experiments the controlled automated reperfusion of the whole body (CARL) protocol with the controlled integrated resuscitation device (CIRD, 1.0 Resuscitec GmbH, Freiburg/Germany) was used (15). ECPR was weaned around 120 min after initiation. This weaning consisted of slowly reducing the flow within 15–20 min until 1.5 L/min. If the pig displayed signs of sufficient circulation (i.e., arterial amplitude above 20 mmHg, MAP above 60 mmHg, and stable lactate measurements) ECPR was discontinued and surgically removed. All post ECPR care and neurologic outcome scoring was comparable to the feasibility test as described above.

### MitoPO_2_ Measurements

The background of PpIX delayed fluorescence measurements is described in detail elsewhere (7). In short, PpIX is the final precursor of heme and is synthesized inside the mitochondria (7). When ALA crème is applied to the skin it enhances the endogenously present PpIX. The PpIX accumulates inside the mitochondria and possesses a triplet state, which reacts strongly with oxygen and therefore it can be used as an intramitochondrial oxygen sensor (7). For this experiment we used the previously described cellular oxygen metabolism monitor (COMET, Photonics Healthcare B.V., Utrecht, The Netherlands) to measure mitoPO_2_ (10). The effect of the ALA crème was tested with an oxygen-consumption measurement performed by applying pressure on the skin sensor. This pressure causes an occlusion of microcirculatory blood flow and therefore oxygen delivery to the mitochondria is stopped, resulting in a decrease of mitoPO_2_ (8, 10). A decrease of mitoPO_2_ to ≤ 5 mmHg and a return to baseline values after release of the pressure on the skin sensor was defined as successful oxygen-consumption measurement. This measurement was performed at least two times before induction of cardiac arrest. During cardiac arrest and during ECPR mitoPO_2_ was measured every minute and, on indication, more often with a maximum frequency of every second for 60 s.

### Other Measurements

Arterial PO_2_ was measured via online blood gas sampling by the CIRD (CIRD-PaO_2_). Systolic, diastolic, and mean arterial pressure were measured invasively via a carotid arterial cannula. For the feasibility test we will plot the mitoPO_2_ and CIRD-PaO_2_ in a graph. All outliers of >200 mmHg will be set at 200 mmHg. The mitoPO_2_ (in mmHg), CIRD-PaO_2_ (in mmHg), and MAP (in mmHg) of the experimental tests will be plotted in graphs from baseline until discontinuation of ECPR flow, which will approximately be at 2.5–3.0 h after initiation of ECPR. Due to the small number of cases, the courses of these measured values cannot be compared using statistical testing. Therefore, the comparing of the graphs will be done by careful visual inspection.

## Results

### Feasibility Test

**Figure 1:**
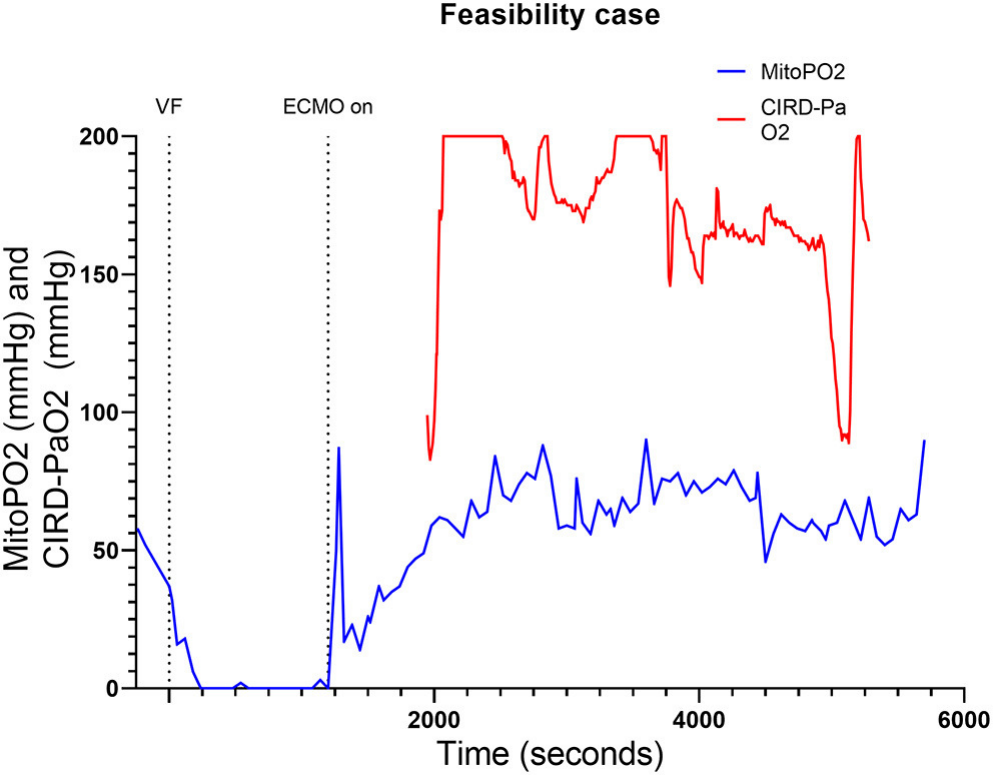
Course of mitoPO_2_ and CIRD-PaO_2_ for the feasibility test. Course of mitoPO_2_ and CIRD-PaO_2_ in mmHg levels over time in seconds. VF, ventricular fibrillation; ECPR, extracorporeal cardiopulmonary resuscitation; CIRD-PaO_2_, arterial partial oxygen pressure measured by controlled integrated resuscitation device; mitoPO_2_, mitochondrial partial oxygen pressure.

The feasibility test we performed, was to find out if skin mitoPO_2_ measurements in a pig during cardiac arrest and ECPR were possible. As shown in Figure 1, mitoPO_2_ of this case dropped after initiation of VF. When ECPR was initiated (after 20 min of VF), an initial spike in mitoPO_2_ followed by a slow upslope was seen. After the initial spike, the level of mitoPO_2_ remained high. This pig survived after ECPR weaning with a NDS at day 1 of 100, at day 2 a NDS of 60, and the following days a NDS of 0. Seven days after the experiment, it was euthanized according to the protocol.

### Experimental Tests

Of the nine experimental tests we performed, four had to be excluded. A detailed description of reasons for exclusion is found in Supplementary Appendix A (10.6084/m9.figshare.13591211). In short, in one experimental test the skin with ALA was exposed to too much light. In two other experimental cases we failed to perform the measurements continuously during the tests. The last experimental case could not be performed due to complication during preparation. Two of these experimental pigs were male and two female.

**Figure 2:**
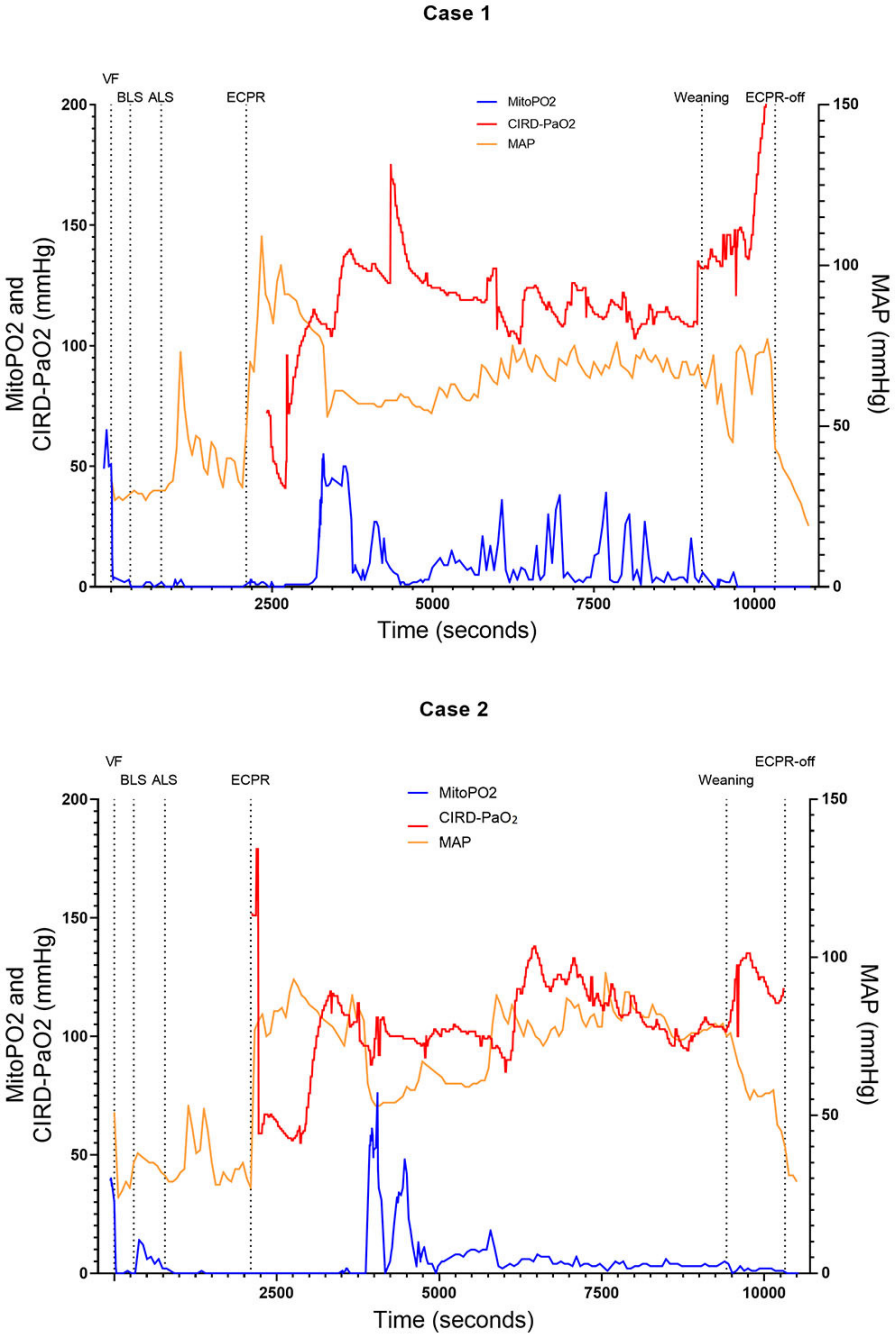
Course of mitoPO_2_, CIRD-PaO_2_, and MAP for experimental tests case 1 and 2. Course of mitoPO_2_ and CIRD-PaO_2_ in mmHg levels at the left *Y*-axis and course of MAP in mmHg levels at the right *Y*-axis all over time in seconds. Case 1 at the above panel and case 2 at the below panel. VF, ventricular fibrillation; ECPR, extracorporeal cardiopulmonary resuscitation; CIRD-PaO_2_, arterial partial oxygen pressure measured by controlled integrated resuscitation device; mitoPO_2_, mitochondrial partial oxygen pressure.

**Figure 3:**
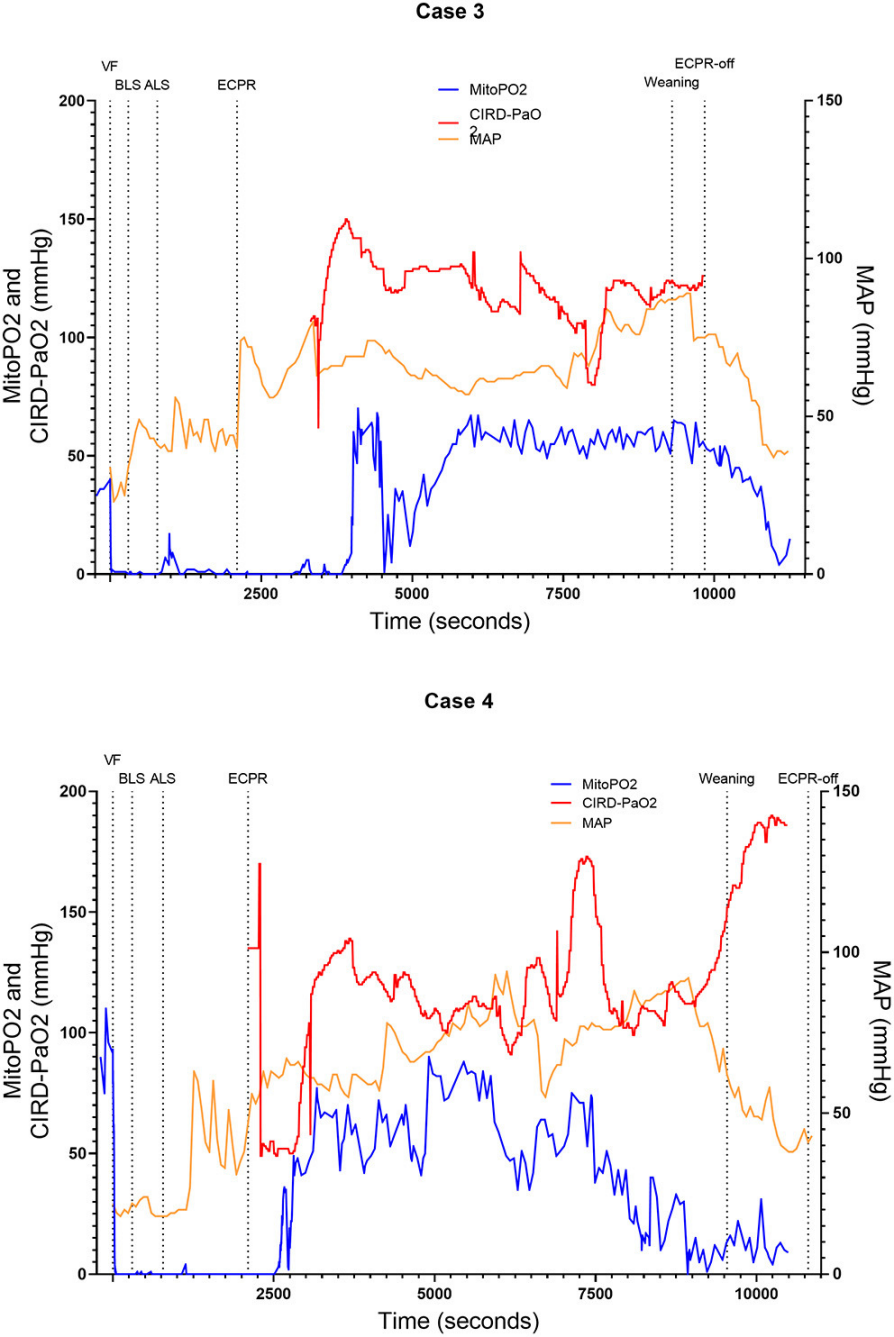
Course of mitoPO_2_, CIRD-PaO_2_, and MAP for experimental tests case 3 and 4. Course of mitoPO_2_ and CIRD-PaO_2_ in mmHg levels at the left *Y*-axis and course of MAP in mmHg levels at the right *Y*-axis all over time in seconds. Case 3 at the above panel and case 4 at the below panel. VF, ventricular fibrillation; ECPR, extracorporeal cardiopulmonary resuscitation; CIRD-PaO_2_, arterial partial oxygen pressure measured by controlled integrated resuscitation device; mitoPO_2_, mitochondrial partial oxygen pressure.

**Figure 4:**
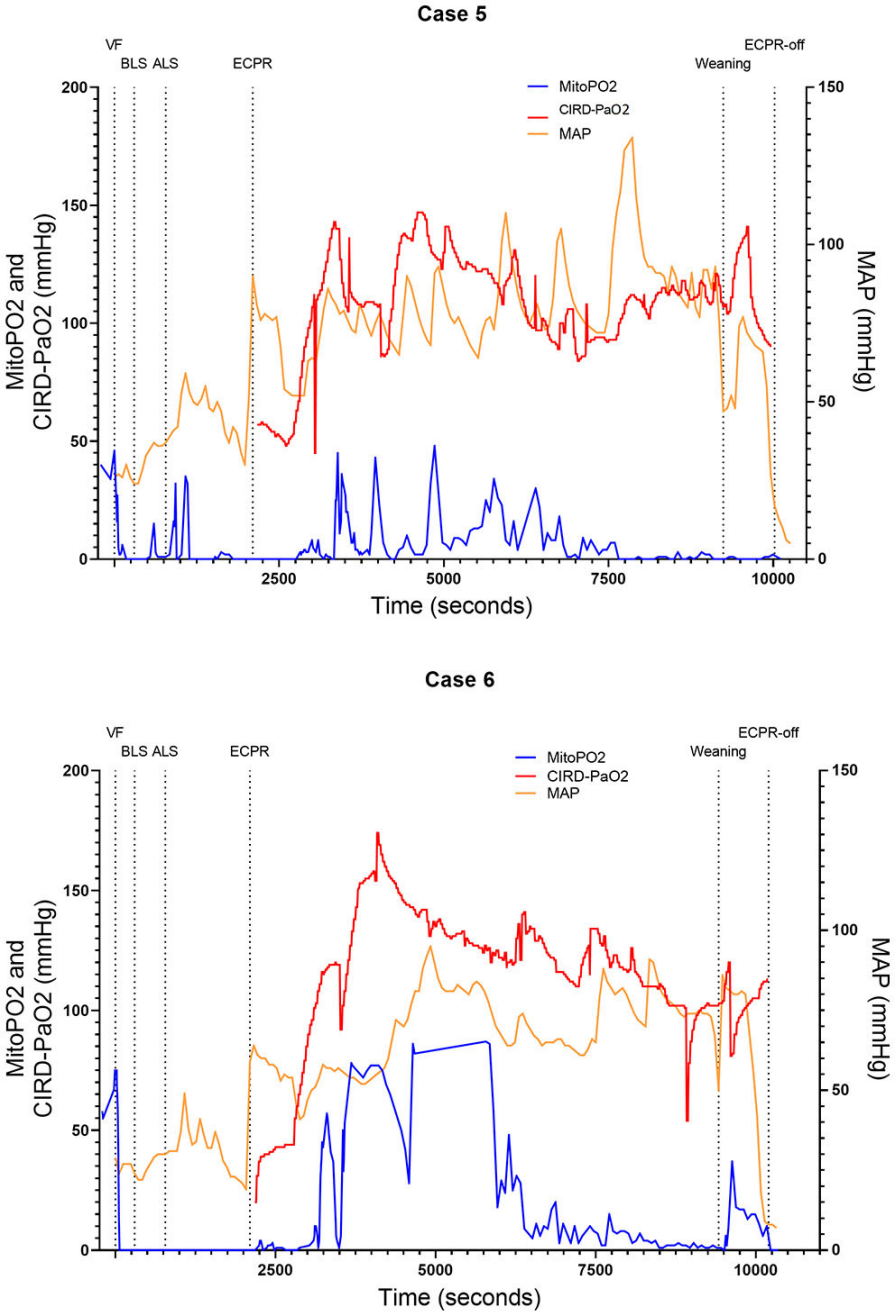
Course of mitoPO_2_, CIRD-PaO_2_, and MAP for experimental tests case 5 and 6. Course of mitoPO_2_ and CIRD-PaO_2_ in mmHg levels at the left *Y*-axis and course of MAP in mmHg levels at the right *Y*-axis all over time in seconds. Case 5 at the above panel and case 6 at the below panel. VF, ventricular fibrillation; ECPR, extracorporeal cardiopulmonary resuscitation; CIRD-PaO_2_, arterial partial oxygen pressure measured by controlled integrated resuscitation device; mitoPO_2_, mitochondrial partial oxygen pressure.

We included six (50.0–70.5 kg) pigs in this experimental test group. The characteristics of the measurements of these pigs are reported in Table 1. Figures 2–4 show the course of mitoPO_2_, CIRD-PaO_2_, and MAP of the six experimental tests in separate graphs, from baseline (just before start of the cardiac arrest) until discontinuation of ECPR flow. In all pigs, directly after initiation of VF, the mitoPO_2_ decreased rapidly. As shown in Table 2, the median time from initiation of VF until a mitoPO_2_ of ≤ 5 mmHg was 29 s (interquartile range, IQR 23–60). The median time from initiation of ECPR until first rise in mitoPO_2_ above 5 mmHg was 1,066 s (i.e., 17 min and 46 s, IQR 900–1,139 s).

**Table 1:** Individual characteristics of measurements in six experimental pigs.

**Case**	**1**	**2**	**3**	**4**	**5**	**6**
Sex	Male	Male	Male	Male	Male	Male
Weight (kg)	50	54	59	62	70.5	70.5
Time: initiation of VF until first low mitoPO_2_ (≤ 5 mmHg) in seconds	26	23	13	31	60	64
Time: initiation ECPR until mitoPO_2_ > 5 mmHg in seconds	1,122	1,829	1,139	481	900	1,008
Correlation of mitoPO_2_ and CIRD-PaO_2_	No	No	Yes	Yes	No	No
ECPR Survival	No	No	No	Yes, 2 days	No	No

*Time initiation of VF until first low mitoPO_2_ is set as the time of initiation of VF until the first mitoPO_2_ measurement of ≤ 5mmHg. VF, ventricular fibrillation; mitoPO_2_, mitochondrial partial oxygen pressure; BLS, basic life support; ALS, advanced life support; ECPR, extracorporeal cardiopulmonary resuscitation; CIRD-PaO_2_, partial oxygen pressure measured by controlled integrated resuscitation device*.

**Table 2:** Summary of characteristics.

**Time: initiation of VF until first low mitoPO_2_ (≤ 5 mmHg) in seconds**	**29 (23–60)**
**Time: initiation ECPR until mitoPO_2_ > 5 mmHg in seconds**	**1,066 (900–1,139)**
**Correlation of mitoPO_2_ and CIRD-PaO_2_**	**2/6 cases (66.7%)**
**ECPR survival**	**1/6 cases (16.7%)**

*The continues variables are presented as medians and interquartile ranges, the categorical variables are presented as number and percentage*.

*VF, ventricular fibrillation; mitoPO_2_, mitochondrial partial oxygen pressure; BLS, basic life support; ALS, advanced life support; ECPR, extracorporeal cardiopulmonary resuscitation; CIRD-PaO_2_, partial oxygen pressure measured by controlled integrated resuscitation device*.

In four of the six experimental tests (case 1, 2, 5, and 6), after initiation of ECPR the initial spike of mitoPO_2_ did not result in persisting high mitoPO_2_ values. In the other two experimental tests (case 3 and 4), after initiation of ECPR the initial spike of mitoPO_2_ was followed by a continuous level of mitoPO_2_ which approached baseline levels. There was no correlation between mitoPO_2_, CIRD-PaO_2_, and MAP in the tests without persisting mitoPO_2_ values. In the two patients with continuous higher levels of mitoPO_2_, comparing the correlation of mitoPO_2_, CIRD-PaO_2_, and MAP is complex. With careful visual inspection, the difference between the three values is smaller and seems somewhat related, especially in case 4. Case 3 could not be successfully weaned from ECPR due to technical problems. Case 4 survived after ECPR weaning. The NDS of this pig was 130 at day 1 and 2 and it was euthanized at day 2 due to humane reasons. All other cases could not be successfully weaned from the ECPR and died at termination of the experiment.

## Discussion

In this study we showed that skin mitoPO_2_ measurements in a pig during cardiac arrest and ECPR are feasible. In all six experimental tests we found a rapid decrease of mitoPO_2_ after initiation of VF and a remarkably delayed increase in the initial measured mitoPO_2_ after reperfusion via ECPR. In four of the six cases no continuously high mitoPO_2_ values were present. However, in two of the cases mitoPO_2_ remained near baseline levels after the initial spike. The course of mitoPO_2_ in these two cases seemed correlated with CIRD-PaO_2_ and MAP. One of these pigs survived, but with an unfavorable neurological outcome until 2 days after the experiment.

The rapid decrease of mitoPO_2_ after initiation of VF was expected to be found, because of the immediate stop of oxygen delivery and uptake due to whole body ischemia. Harms et al. (8) showed a rapid decrease of mitoPO_2_ as soon as oxygen delivery to the skin is stopped by performing local pressure to the skin sensor, which occludes the microvessels, in a rat experiment. Ubbink et al. (10) repeated this test in humans and they also found a rapid decrease in mitoPO_2_ when applying local pressure. After initiation of VF in our tests, systemic blood flow stops and therefore there will be a rapid decrease in oxygen delivery. This disappearance of blood flow and oxygen delivery acts the same as the oxygen consumption tests performing pressure to occlude the microvessels. This demonstrates the close relationship of tissue perfusion and tissue oxygenation.

The delay of increase in mitoPO_2_ after initiation of ECPR we found is most probably due to adrenaline administered during ALS according to the resuscitation guidelines. The possible effects of medication on mitoPO_2_ has been shown before. Ubbink et al. (10) showed, as an incidental finding, that the initial vasoconstriction caused by clonidine decreased the skin mitoPO_2_ values. There was no effect on capillary venous oxygen saturation, however the effect on mitoPO_2_ and flow remained present for around 15 min (10). Adrenaline stimulates the α1-receptors, among others, which causes vasoconstriction and centralization of blood flow (16). The effect of adrenaline on mitoPO_2_ values has not been shown before. However, Fries et al. (17) showed that administration of adrenaline caused a decrease in capillary blood flow which persisted after the achievement of return of spontaneous circulation (ROSC). Therefore, administration of adrenaline might explain the delay of increase in skin mitoPO_2_ values.

Another explanation for the delay of increase in mitoPO_2_ after initiation of ECPR could be centralization, where the skin as an end organ will be the last organ to regain flow. After initiation of ECPR, the macrocirculation (MAP and blood flow) is restored immediately. However, the exact timing of restoration of the microcirculation in different tissues is unknown. In cardiac arrest, arterial blood flow decreases to zero and during CPR it will remain lower than normal (18). Therefore the reduced stimulation of the arterial baroreceptors will activate the sympathetic system (19). This sympathetic system will centralize the blood flow and causes vasoconstriction to preserve heart and brain function (19). A hypothesis could be that when ECPR is initiated this vasoconstriction is present and only when the other vital organs are perfused, the perfusion of the skin will recover.

In four of the six experimental tests, the delay of increase in mitoPO_2_ despite systemic reperfusion suggests irreversible damage to tissue perfusion, tissue oxygenation, or oxygen transport to the mitochondria. In these four tests, after the initial spike of high mitoPO_2_ following ECPR initiation, no continuously high mitoPO_2_ values were measured until the end of the experiment. In the two tests with recovery of the skin mitoPO_2_ values, recovery of the tissue perfusion, tissue oxygenation, and oxygen transport to the mitochondria takes place. When high mitoPO_2_ values were measured after the initial spike, the values remained high. The inadequacy of tissue oxygenation and long ischemic period could eventually lead to a microcirculatory shut down. A global shut down in cases of ischemia was already reported before (20). After an ischemic episode, the microcirculation respiration can recover to a certain extent, depending on the duration of ischemia and level of reperfusion (20). Ruggieri et al. (21) stated that, after severe ischemia some muscle cells in the heart can be irreversibly damaged and demarcation will occur, while the incompletely effected tissue can recover partly. The difference in mitoPO_2_ course in the six experimental tests we performed could be explained by irreversible vs. incomplete effected tissue. We hypothesize that the quicker mitoPO_2_ rises could be explained by less ischaemic cellular damage which could cause sooner re-activation of the mitochondrial function and therefore less overall ischaemic/reperfusion damage.

MitoPO_2_ can be interpreted as determinant of the microcirculatory function. In order to regain oxygen levels into the mitochondria after cardiac arrest, tissue perfusion and tissue oxygenation have to be present. This tissue perfusion and oxygenation are largely influenced by an intact microcirculation. In case mitoPO_2_ is detected, it can be expected that microcirculation has at least partly been recovered. Bodmer et al. (22) performed a study measuring the microcirculation and mitoPO_2_ in the liver simultaneously. They found only a small difference of PO_2_ in the microcirculation and mitoPO_2_. Mik et al. (9) recently stated that average mitoPO_2_ appears to be close to microvascular PO_2_. MitoPO_2_ measured by PpIX delayed fluorescence provides an estimation of microvascular PO_2_ and therefore an it can be interpreted as determinant of the microcirculatory function.

The added value of monitoring microcirculatory function could be relevant in ECPR procedures, as the correlation between the mitoPO_2_ and MAP is not consistently present in this study. In contrast to the abovementioned delay in recovery of tissue oxygenation due to an impaired microcirculatory function, the macrocirculation (MAP and blood flow) was restored immediately upon initiation of ECPR. This discrepancy between mitoPO_2_, as a determinant of microcirculatory function, and MAP, as a determinant of macrocirculation, shows the importance of monitoring this microcirculatory function. Yu et al. (23) compared the microcirculation and macrocirculation and showed an inconsistent relation of microcirculation (i.e., brain and brachioradial muscle) and macrocirculation (i.e., MAP) in pigs with cardiac arrest (23). Fritz et al. (24) performed ECPR in pigs, they found no differences in microcirculatory flow index at initiation and after 6 h of ECPR comparing the group treated with standard MAP to the group treated with high MAP. However, they did not directly investigate the relation between continuous microcirculatory function and MAP. In addition, two other studies compared microcirculation and macrocirculation in patients with cardiogenic shock treated with veno-arterial ECMO (VA-ECMO) (25, 26). Yeh et al. (26) showed no differences in early MAP for survivors and non-survivors, while early microcirculation was higher in survivors than in non-survivors. Chommeloux et al. (25) showed in successfully weaned patients despite normal MAP, normalization of microcirculatory values took 48 h after initiation of VA-ECMO. In order to apply personalized medicine and therefore a possible increase in the accuracy of treatment, monitoring of microcirculatory function should be the added to monitoring of macrocirculation.

In addition, the survival chance in pigs with continuously high mitoPO_2_ is probably higher than in pigs without continuously high mitoPO_2_. One of the two cases with continuously high mitoPO_2_ survived this experiment. The other one died because of technical failure. In the experiments with pigs without continuously high mitoPO_2_, none of them survived. No previous studies aimed on the course of mitoPO_2_ measurements in ECPR in relation to survival outcome. However, Fries et al. (17) found less increase in microcirculation (i.e., capillary flow) in animals that failed resuscitation in a model with chest compressions. Furthermore, within 5 min of ROSC, microcirculation returned only within 20% of baseline values (17). This could point to the possible additional value of monitoring microcirculatory function during CPR, during ECPR, and after ROSC.

This study has several limitations. First, our sample size is small and of the experimental measurements only one pig survived. Therefore, we could not perform statistical tests to identify the correlation of the course of mitoPO_2_ and survival or favorable neurological survival. Possible hypotheses for the low successful weaning numbers in this studies are described in the Supplementary Appendix B. Second, because of the preparation time of the ALA crème, mitoPO_2_ measurements cannot be used during cardiac arrest and within the first hours after ECPR initiation in humans. In order to test our hypotheses and get more understanding of the pathophysiology, future experimental research should aim at the correlation of the course of mitoPO_2_ and survival. Also, the course of mitoPO_2_ comparing conventional CPR with ROSC and ECPR cases could extend the existing knowledge. Another topic which needs to be investigated in future research is if, in humans treated with ECPR, monitoring the microcirculatory function is a more accurate target than monitoring the macrocirculation in order to apply a more individually based treatment. If a method is found to shorten the time needed for ALA-induced PpIX enhancement within cells (e.g., with intravenous administration), mitoPO_2_ measurements during ECPR could be also performed in humans.

## Conclusion

This experimental pilot study shows that continuous measurements of skin mitoPO_2_ in pigs treated with ECPR are feasible. The delay in initial mitoPO_2_ and discrepancy of mitoPO_2_ and MAP in our small sample study could point to the possible value of additional measurements besides MAP to monitor the quality of tissue perfusion during cardiac arrest and ECPR.

## Data Availability Statement

The original contributions presented in the study are included in the article/Supplementary Material, further inquiries can be directed to the corresponding author/s.

## Ethics Statement

The animal study was reviewed and approved by Committee for Ethics of the University Hospital Freiburg, Freiburg, Germany (No. G-15/148).

## Author Contributions

LM participated in the study design, acquired, analyzed, interpreted the data, and drafted and revised the manuscript. J-SP helped acquiring, analyzing, and interpreting the data and was a contributor in revising the manuscript. MW and DD helped interpreting the data and were major contributors in revising the manuscript. CU participated in the study design, helped interpreting the data, and was a major contributor in revising the manuscript. EM and GT participated in the study design, helped interpreting the data, and were contributors in revising the manuscript. SB helped acquiring the data and was a contributor in revising the manuscript. DG participated in the study design and was a contributor in revising the manuscript. DR majorly contributed to the conception of the study, participated in the study design, helped interpreting the data, and was a major contributor in revising the manuscript. All authors read and approved the final manuscript.

## Conflict of Interest

DR declares having received speaking fees from Xenios GmbH and HillRom GmbH. EM is listed as inventor on patents related to mitochondrial oxygen measurements held by the Academic Medical Center Amsterdam and the Erasmus Medical Center Rotterdam, the Netherlands. EM is founder and shareholder of Photonics Healthcare, a company that holds exclusive licenses to these patents and that markets the COMET system. DG is a member of the medical advisory board of Xenios GmbH and received travel expenses and speaker fees from Xenios and Maquet GmbH. GT is shareholder of Resuscitec GmbH. The remaining authors declare that the research was conducted in the absence of any commercial or financial relationships that could be construed as a potential conflict of interest.

## Publisher's Note

All claims expressed in this article are solely those of the authors and do not necessarily represent those of their affiliated organizations, or those of the publisher, the editors and the reviewers. Any product that may be evaluated in this article, or claim that may be made by its manufacturer, is not guaranteed or endorsed by the publisher.

## Abbreviations

ALA: 5-Aminolevulinic Acid Hydrochloride
ALS: Advanced Life Support
BLS: Basic Life Support
CARL: Controlled Automated Reperfusion of the Whole Body
CIRD: Controlled Integrated Resuscitation Device
CIRD-PaO_2_: Partial oxygen pressure measured by Controlled Integrated Resuscitation Device
CPR: Cardiopulmonary Resuscitation
COMET: Cellular Oxygen Metabolism Monitor
ECPR: Extracorporeal Cardiopulmonary Resuscitation
MAP: Mean Arterial Pressure
MitoPO_2_: Mitochondrial partial oxygen pressure
NaCl: Sodium Chloride
NDS: Neurologic Deficit Score
PO_2_: Partial Oxygen Pressure
PpIX: Protoporphyrine IX
ROSC: Return Of Spontaneous Circulation
VA-ECMO: Venoarterial-Extracorporeal Membrane Oxygenation
VF: Ventricular Fibrillation.
