# Supplementary material for: Monitoring Mitochondrial Partial Oxygen Pressure During Cardiac Arrest and Extracorporeal Cardiopulmonary Resuscitation. An Experimental Pilot Study in a Pig Model
Source: Front Cardiovasc Med. 2021 Oct 25;8:754852. doi: 10.3389/fcvm.2021.754852 (PMC8572977; doi:10.3389/fcvm.2021.754852)
Supplement: Supplementary file 1 [file Data_Sheet_1.docx]

Supplementary Material

**Appendix A; excluded experiments**

In the first experimental case, the spot we prepared for the measurements, was only protected to light by some operation covers. Every value we measured during the complete three hours of testing was <5mmHg, most probably because of the exposure to light.

For the next two failed experiments we protected the measuring spots with aluminium foil and during the test we covered it with an aluminium tray covered with black tape. Of the two prepared spots, one kept covered with aluminium foil in order to keep it protected to light. After initiation of ECMO, there was an unexpected delay in mitochondrial PO2 (mitoPO_2_) recovery. Therefore, the hypothesis was that again, there had been exposure to light. We tried to retrieve results by uncovering the remaining (previously covered) measuring spot. During this switch between the two measuring spots, we retrieved mitoPO_2_ measurements. However, it was not clear if exposure to light had influenced the measurements and what was the exact timing of the spike of mitoPO_2_. Therefore, we determined to exclude these measurements. After these tests, we realised that it could be part of the experimental outcomes and not due to the oxygen exposure. The next experiment we performed, we continued the measurements manually at one spot until the first mitoPO_2_ was detected. As in the measurements before we found a delay and all other tests were performed at one spot as much as possible and at least until the first mitoPO_2_ value after initiation of ECPR.

Last, we had to exclude one experimental case because in the preparation phase, while attempting to place a intracranial pressure catheter, a bleeding of the olfactory artery occurred. Unfortunately this could not be resolved in time and the experiment had to be cancelled.

**Appendix B**

In this study, in most of the cases weaning was not successful. Due to the small sample size and low successful weaning numbers, causes of successful and not successful weaning are unknown. However, we provide some hypotheses for the low successful weaning numbers. First, the use of the Corpuls as mechanical compression device is not made for the thoracic shape of the pigs. It might cause damage causing pneumothorax or bleedings, or be insufficient in retrieving some output. Second, the duration of ECMO support is very short and weaning has to take place shortly after ischaemia and reperfusion. It could be that with a longer ECMO run, more recovery of cardiac function could take place. Third, during CPR, a high dosage of catecholamine is used (8-9mg), which could have influenced cardiac function.
